# DICOM Structured Reporting and Cancer Clinical Trials Results

**DOI:** 10.4137/cin.s37032

**Published:** 2007-05-12

**Authors:** David A Clunie

**Keywords:** DICOM, Structured Reporting, Clinical Trials

## Abstract

The use of biomarkers derived from radiological images as surrogate end-points in therapeutic cancer clinical trials is well established. DICOM is the ubiquitous standard for the interchange of images for both clinical use as well as research. It also has capabilities for the exchange of image-related information, including categorical and quantitative information derived from images. The use of DICOM Structured Reporting for the encoding and interchange of clinical trial results in a standard manner is reviewed.

## Background

### Biomarkers and endpoints

Human therapeutic clinical trials for cancer have traditionally used survival as the primary endpoint. Increasingly, in order to reduce the cost of clinical trials as well as to accelerate the approval of new agents, alternative (“surrogate”) end points are used (Johnson, 2003). Response assessment determined from radiological studies, as opposed to clinical evidence, is frequently used. For example, in order to evaluate the response of solid tumors to therapy, the size of lesions measured from Computerized Tomography (CT) scans is often used.

Experience has been formalized into evaluation criteria that have been refined over time and adapted to improving technology. Various groups have defined and published criteria that are now well established, such as the World Health Organization (WHO) criteria (WHO, 1979) and the Response Evaluation Criteria In Solid Tumors (RECIST) (Therrase, 2000). Other criteria based on similar principles have been developed for other types of tumors. These traditional criteria combine relatively easy-to-measure quantitative information about tumor size, such as the longest single dimension on an axial slice for measurable disease (“target” lesions), with qualitative information about disease that is deemed not to be measurable (“non-target” lesions, or new lesions), in order to algorithmically derive a categorical assessment of response. Usually, these categories are Progressive Disease (PD), Stable Disease (SD), Partial Response (PR) or Complete Response(CR).

Image acquisition technology, image processing techniques and computing power are all improving. Modalities other than CT, such as Magnetic Resonance Imaging (MRI), and Positron Emission Tomography (PET) are now widely available even for large international multi-center clinical trials that may be conducted in second and third world countries. The prospect exists for developing alternative biomarkers either that are a better estimate of tumor size, such as a three dimensional volume from multi-detector CT images, or that measure another parameter of a tumor that is quantifiable by imaging. Examples of the latter include dynamic contrast enhancement on CT or MRI as a surrogate for vascularity or permeability, CT density as a surrogate for change in tissue composition, or fluorodeoxyglucose (FDG) PET Standardized Uptake Values (SUV) as a surrogate for metabolic activity. The use of such novel biomarkers for regulatory approval is expected to increase as they are validated as surrogate endpoints. In the United States, the Food and Drug Administration (FDA) is undertaking a project to evaluate potential biomarkers and validate them as surrogate endpoints (FDA, 2006).

### Independent review process and standardization

If a trial were to be conducted by a single team at a single site, the manner in which measurements derived from images were recorded would be of little consequence, and could be performed in a proprietary manner. However, the majority of cancer trials are not only multi-center trials, but in general involve blinded evaluations performed by an independent organization or dedicated group of observers, in order to eliminate bias and reduce variance. This is true both for academic and commercial regulatory approval trials (FDA, 2004; FDA, 2005). Since the images may be gathered from multiple sites, and the quantitative measurements and qualitative analysis may be made by multiple expert observers, a need is created to interchange the images and the evaluations, preferably in a standard form. Indeed, further opportunities for standardization arise at multiple steps in the process, since it is common for different organizations to:

- design and sponsor the trial,
- perform the imaging,
- take on the responsibility for the clinical care of the patients,
- handle the logistics of gathering and checking the quality of the images,
- perform the expert read, check the quality of the results,
- perform the statistical analysis, and submit the electronic report and accompanying information for regulatory approval.

For all radiological imaging modalities, there is no question as to what the standard format for transfer between the imaging centers and the central review facility should be. The Digital Imaging and Communications in Medicine (DICOM) standard, first published in 1993 but regularly updated and revised (DICOM, 2007), has rapidly become the only standard in widespread use for radiological imaging. It is also used for many other types of medical imaging. All modern digital radiology imaging equipment is available with a DICOM interface and older devices are typically upgraded or retrofitted. DICOM digital images for clinical trials are transferred either across a wide area network (WAN) using a secure connection, or more commonly, a DICOM Compact Disc (CD) or Magneto-optical Disk (MOD) is sent by courier. When only film is available, for clinical trials it is typically digitized with medical-grade scanning equipment and encoded in DICOM form.

Though the interchange of digital images is a problem that has essentially been solved with DICOM, there are few widely adopted standards for the encoding of image-related clinical trial information. Specifically, there are no standards for the encoding of information that accompanies the image from the site, which might describe details of the acquisition technique or process that might not be encoded in the image headers by the modality. There are also no standards for the encoding of the quantitative or categorical information derived during the reading or analysis process. The primary focus of this paper will be on the latter, that is, the encoding of result information, There are other standardization activities related to the encoding of electronic case report forms, such as by the Clinical Data Interchange Standards Consortium (CDISC), the Integrating the Healthcare Enterprise (IHE) Retrieve Form for Data Capture (RFD) profile (IHE, 2006a) and the Case Report Form (CRF) special interest group of the Cancer Biomedical Informatics Grid (caBIG).

There are two general approaches to encoding the result information. First, the primary consumers of the response assessments will likely be those responsible for data management and statistical analysis, who are primarily interested in the aggregated response information about each subject at each time point, and are generally not interested either in details of the size or characteristics of individual lesions. Second, there are those who are responsible for the review of the individual lesions together with the images, either for quality control, adjudication in the case of multiple readers with conflicting response assessments, or review by qualified experts at the behest of regulatory authorities.

The former constituency is usually satisfied with tabulated data exchanged via mechanisms common in the data management community, such as fixed width column or delimiter separated text files or various proprietary spreadsheet or statistical package formats, with the appropriate transfer mechanisms in place to ensure security and integrity. The interchange files are usually generated by databases, exchanged, and loaded into other databases, the schemas of which are tabular in nature and designed according to the needs of the type of the trial. The CDISC Study Data Tabulation Model (SDTM) is one approach to standardizing this type of non-image related aggregated data (CDISC, 2004).

The latter group, however, need information that is usable by medical image display software, which is more granular in nature, and is less easily tabulated. Specifically, the coordinates of the endpoints of distance measurements, or the outlines of lesion boundaries are needed for display on the images, together with the quantitative, descriptive and categorical information, such as lesion size, anatomical location and response assessment. It is for this use case that the remainder of this paper will explore the use of other mechanisms available in DICOM, specifically the applicability of DICOM Structured Reports (SR).

## DICOM Structured Reports

### Definition, history and adoption

The DICOM Structured Report (SR) was designed to be a “self-describing information structure” that could be “tailored to diverse clinical observation reporting applications by utilization of templates and context-dependent terminology” (Bidgood, 1997). A DICOM SR document consists of an ordinary DICOM “header” containing demographic and identification information, accompanied by a “content tree” that consists of a recursive structure of name-value pairs. Extensive use is made of codes rather than plain text, numeric measurements, and references to images and coordinates of regions on those images.

Beyond the realm of clinical trials, there are many reasons to encode structured, quantitative and coded information related to images. Indeed the ordinary human-readable radiology report, authored by radiologists for the consumption by an ordering physician, is often implicitly structured, by section headings, may contain quantitative information that has been measured and recorded manually, and frequently contains codes or keywords, for reimbursement purposes as well as for automated text generation from macros. One of the initial goals for the development of the DICOM Structured Report (SR) capability was to encode such reports, in a form that would allow information to be extracted more readily than from a paper printed report, or an unstructured plain text format. It was hoped that a transition to such an encoding would enable indexing and selective retrieval, without having to resort to Natural Language Parsing (NLP) (Langlotz, 2002). However, in practice, there seem to be many barriers to encoding structure rather than plain text. Principally these are the additional effort required to author a structured document as opposed to free speech dictation, and the lack of a means to disseminate structured documents beyond the radiology environment, since plain text remains the *lingua franca* of healthcare information systems for the time being. This is true despite the fact that there is some evidence that would suggest that referring physicians prefer itemized rather than prose reports (Naik, 2001).

Fortunately, greater success has been achieved with the encoding of machine-generated structured content. There are two general categories of such information, that generated by human operators using imaging equipment, such as distance and velocity measurements made during vascular, cardiac or obstetric ultrasound, and that generated by automated analysis of images, including so-called Computer Assisted Detection (CAD) or Computer Assisted Diagnosis (CADx). Both types of use fall into the general category of creation of “evidence documents”, part of the content of which is often subsequently extracted and included in human-generated reports that supply the interpretation of the findings. The DICOM SR framework has proven ideal for the encoding, interchange and persistent storage of such evidence documents, as was intended. This is largely because both the acquisition equipment and the image reporting equipment already support DICOM encoding and services in order to be able to exchange images, and it is less additional effort to extend the DICOM implementation than it is to develop some novel method. DICOM SR is rapidly supplanting various proprietary exchange mechanisms in this role, not only for radiology, but for cardiology as well (IHE, 2005).

The requirements for encoding of structured, quantitative and coded information derived from images as evidence documents would seem very similar to those required for encoding clinical trial results as described earlier. Subsequent sections of this paper will describe in detail how such information is encoded, and what additional steps are necessary to ensure reliable interchange between clinical trials systems.

### Content of a DICOM structured report

The manner in which the content tree of a DICOM SR is encoded is illustrated by way of an example of a Mammography CAD SR. The CAD task is a clinical task that most closely approximates the encoding of clinical trial results, and mammography is the most widespread form of CAD currently deployed in commercial medical image devices. Indeed, with the rapidly increasing deployment of digital mammography as a consequence of the results of the Digital Mammographic Imaging Screening Trial (DMIST) (Pisano, 2006), it is likely that the ability to display DICOM Mammography CAD SR will be required in all digital mammography workstations, as is advocated by the Integrating the Healthcare Enterprise (IHE) Mammography Image Profile (IHE, 2006b).

**Figure 1:**
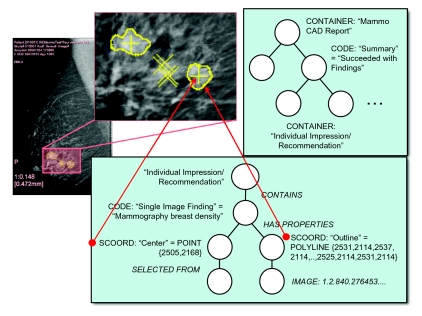
Mammography CAD findings on a single image view, the sub-region containing the findings magnified, and the corresponding top-level and individual finding sub-trees encoded in a DICOM Structured Report.

Findings on digital mammograms include micro-calcifications, masses and architectural distortion that may have morphological characteristics indicative of malignancy as opposed to benignity. The machine interpretation task also may take into account change over time, an important factor in assessment of malignancy; though this is currently beyond the state of the art of CAD devices and is usually performed by a human, the DICOM SR template allows for the encoding of this information. Figure 1 illustrates several findings on a single view of a digital mammogram, and the tree structure that is encoded in the corresponding DICOM SR.

A DICOM SR consists of a tree of content rooted at a single node, which contains the title of the report, in this case a coded name that means “Mammography CAD Report”. For illustrative purposes, the codes need not be elaborated, but do not get the mistaken impression that the free text may be used for the concept names for it may not. The root node may have one or more child nodes, each of which may have further sub-ordinate nodes, recursively, forming a tree. In fact, since nodes may reference other non-child nodes in certain cases, the structure may potentially be an acyclic graph rather than a simple tree.

Each node is referred to in the standard as a “content item”. Each content item is of a particular type of which a limited number are defined in the standard. Depending on the type, the content item may be composed of a name only (in the case of a CONTAINER type) or a name-value pair (for all other types). As mentioned, the name is always coded; the value may be a code, free text, or some more complex set of attributes depending on the type. In the example in Figure 1 are illustrated the following content item types:

- CONTAINER, which has no value per se but serves only to contain its children, and has a coded name, which may, for example, be considered analogous to a section heading;
- CODE, which describes a coded concept and its coded value, for example in this case there is a “finding” that is a “breast density” (i.e. a mass);
- SCOORD, which describes a coded concept, in this case the “center” and the “outline” of the finding, a form, in this case a single point or multiple points composing a series of line segments, and the spatial coordinates (location of the pixels) on the corresponding image;
- IMAGE, which references a specific image by its unique identifier

Though not shown in this example, other value types of particular relevance to the encoding of results of clinical trials include:

- NUM, which describes a numeric finding with a coded concept, a numeric value and coded units of measurement;
- TEXT, which describes a finding with a coded concept but a plain text value;
- DATE, TIME and DATETIME, which describe a coded concept and a date or time value;
- PNAME, which describes a coded concept and a person’s name, such as who made an observation.

The arcs joining parent and child content items are tagged with a relationship type. There are only a limited number of relationship types in DICOM SR. They may serve to define the meaning of the relationship between a parent and its child. For example, children with a HAS PROPERTIES relationship may describe the attributes of a parent, or the evidence from which an assertion has been derived may be described with an INFERRED FROM relationship. Alternatively, the relationship type may serve a structural purpose. For example, a CONTAINER content item can only have a CONTAINS relationship with its children, and SCOORD content items always have a SELECTED FROM relationship with a single child. An exhaustive list of the available content item types and relationship types can be found in the standard itself (DICOM, 2007) or a textbook on the subject (Clunie, 2001).

### Coded terminology

A core feature of DICOM SR is the use of codes from controlled vocabularies that are used in other healthcare domains, in order to enable broader use of information extracted from DICOM documents. A general process used when new concepts are defined during the development and maintenance of DICOM, is to first examine existing external terminologies to see if the concept is already available, if not, to offer the concept and its definition to an appropriate authority, such as SNOMED, and only if they do not choose to adopt it to define a DICOM-specific concept. DICOM does maintain its own terminology as Part 16 of DICOM for those concepts that are imaging-specific (DICOM, 2007), though it is expected that an increasing proportion of those will in future be defined by a new effort by the Radiological Society of North America (RSNA), the Lexicon for Uniform Indexing and Retrieval of Radiology Information Resources (RadLex) (RSNA, 2003).

DICOM requires that a human-readable meaning (display name) always be encoded for any code chosen from a controlled vocabulary. The rationale is that the recipient may not have access to a dictionary or service in which to look up the code yet may need to render it. Codes are therefore always a triplet of coding scheme designator, code value, and a human-readable code meaning. An example of such a triplet is (F-01766,SRT,“Punctate Calcification”). The coding scheme designator defines the source of the codes. This example uses a concept that is described in the American College of Radiology (ACR) Breast Imaging Reporting and Data System (BI-RADS) (ACR, 2003). The concept was added to the Systematized Nomenclature of Medicine (SNOMED) (SNOMED, 2006) at the request of DICOM, for the purpose of encoding mammography reports and CAD results, hence “SRT” is used as the coding scheme designator.

### Shorthand

Rather than continue to illustrate examples graphically, an informal shorthand textual notation is introduced that will be used in the rest of this paper. This short hand will illustrate levels of nesting by indentation and a preceding greater than symbol, explicitly show relationship types and content item types, indicate coded information in parentheses and separate names from values with equals signs. The example of the finding illustrated in Figure 1 could be written:

<contains CODE: (,,“Single Image Finding”)=

(,,“Mammographic breast density”)>

> <has properties SCOORD:(,,“Center”)=

(POINT,2505,2168)>

>> <selected from IMAGE: =

(MG Image,“1.2.840…”)>

This is not the form in which DICOM SR documents are actually interchanged; a binary list of tag-value data element is encoded, in the same manner as images are stored and transferred.

When more complex examples are shown and it is not necessary for the purpose of illustration to describe the relationship types explicitly, they will be elided for compactness, as in the following example:

<CODE:(,,“Single Image Finding”) =

(,,“Mammographic breast density”)

>> <SCOORD:(,,“Center”)=(POINT,2505,2168)>

>> <IMAGE: = (MG Image,“1.2.840…”)>

The coding scheme and code value will also be omitted and only the meaning of codes shown. For example, the concept code (,,“Punctate Calcification”) would more completely be described as (F-01766,SRT,“Punctate Calcification”).

### Templates

It should be apparent from the foregoing that the DICOM SR infrastructure is extremely generic, and can be used to encode almost any form of information. This is a strength in terms of flexibility and extensibility, but also a weakness, in that it requires the recipient of the document to deal with excessive generality. DICOM SR is analogous to eXtensible Markup Language (XML) in this and other respects. In the XML world, this generality is constrained by Document Type Definitions (DTDs) and Schemas that specify restrictions on the general form for specific purposes. The equivalent mechanism in DICOM is the SR template.

Each SR template is defined in the DICOM standard and developed by consensus of expert vendor and user representatives to satisfy a particular clinical use case. These templates are normative in that they define the structure of an SR document for a particular purpose, the types of content items and relationships, the codes for concepts and values that shall be used, and the minimum amount of content that is required, together with additional optional content.

Templates are defined at two levels:

- root templates, which are those that define the top-level structure of the entire document from the root node, such as the Mammography CAD Document Root Template;
- sub-templates, which define sub-trees or patterns within the document for a specific purpose, such as the Mammography CAD Single Image Finding Template.

Templates are documented in such a manner that either the form of individual content items is described, or other templates are used as macros by inclusion, potentially recursively. Sub-templates may be further classified in two types:

- those that are specific to a particular use-case and used only by inclusion in root templates or their direct descendants, for example the Mammography CAD Single Image Finding Template;
- those that are intended to be generic and reusable as a component for many use-cases, such as the Linear Measurement Template.

An example of the tabular representation of a simple template is illustrated in Table 1, which is the Linear Measurement Template. Note that it is important to distinguish the description of a template from an instance of a content tree generated in conformance with a template. In the interest of brevity, further discussion of templates will largely be illustrated by examples of instances rather than the templates themselves.

### Value sets and context groups

A template may define either a single coded term for a concept name of a particular content item, or a set of coded terms that shall be used. Further, when the value of a content item is coded, or the units of a numeric measurement are specified, again, either a single term or a set of terms is specified. Such a value set containing a list of coded terms is referred to in DICOM as a context group. The name “context group” is used since the original intent was that the meaning of the terms would be conditioned by the context in which they were used (Bidgood, 1997), though in contemporary usage this approach is deprecated and the coded terms themselves are expected to be sufficiently well defined as to be self-describing, regardless of context.

Like templates, context groups may be defined that are specific to a particular template for a particular use-case, or may be general purpose and re-usable, as is the case with the Linear Measurement and Units of Linear Measurement context groups illustrated in Tables 2 and 3. Note that any particular context group may contain coded concepts drawn from multiple different coding schemes.

### Template instantiation

Templates define limits on how an entire SR tree or some part of an SR tree may be constructed. Let us consider an example of how to instantiate a particular template, in this case, the Linear Measurement template.

A user or a device has identified two points on an image that constitute the ends of a line representing the long axis of cross-section of a mass. The information available to encode consists of:

- the unique identifier of the image on which the measurement was made,
- the row and column coordinates of the two end points of the line
- the length of the line in millimeters within the patient, derived from the length in pixels and the known horizontal and vertical size of each pixel (as encoded in the DICOM image header by the acquisition device).

Given the general DICOM SR infrastructure, there are a myriad ways in which such information could be encoded. The Linear Measurement template serves to constrain the possibilities to a single pattern, and would be instantiated for our example as follows:

<NUM: (G-A185,SNM3,“Long Axis”) = “13.7”

(mm,UCUM,“millimeter”)>

> <has properties SCOORD:(121055,DCM,“Path”)=

(POLYLINE,133,264,124,273)>

>> <selected from IMAGE: =

(CT Image,“1.2.840…”)>

The value of such a template is that all receiving applications that are aware of this template can find and display such measurements and their locations on an image in a predictable manner, or extract the derived information for tabulation or statistical analysis.

## Templates For Clinical Trials

### Introduction

By now it should be apparent that the DICOM SR infrastructure, template and coded terminology mechanisms are well suited to the interchange of clinical trial results derived from images and associated information.

The infrastructure provides the core encoding capabilities for recording quantitative and categorical information and its relationship to images and locations within those images, together with a hierarchical organizational structure. Clinical applications have driven the development of standard reusable sub-templates that may be used to assemble templates specific for clinical trials. The general structure of the root templates standardized for CAD applications provide a basis from which to design new templates for similar use-cases.

It remains to:

- identify the requirements that are specific for each type of clinical trial,
- select the appropriate existing DICOM SR templates and develop new ones where gaps in the existing standard are identified,
- identify appropriate sets of coded terms,
- refine existing terms or define new terms where necessary,
- assemble these components into root templates that form the basis for interchange and interoperability.

This paper is not the place in which to develop such a standard, but in general terms this section will explore the requirements, gaps and propose solutions in broad terms. It remains for the professional societies, other standards bodies and government organizations to propose and develop the new templates in conjunction with the DICOM organization. For example, DICOM has a working group specifically dedicated to clinical trials, working group 18, which has been quiescent since completing its last work item, but which could be reactivated to perform the task. The National Cancer Institute (NCI) within the In Vivo Imaging Workspace of the Cancer Bioinformatics Grid (caBIG) project has identified a specific project related to the encoding and interchange of Standard Image Annotation and Markup (SIAM), which will be encouraged to extend DICOM SR rather than develop an incompatible standard *de novo*.

### Structure

Since each DICOM SR instance is a persistent document in its own right, uniquely identified and potentially referenced from other objects, fundamental design questions arise as to the scope of the information encoded in a single instance. Specifically, shall each instance encode the results of a single subject or multiple subjects, those of a single time point or multiple time points, a single radiological examination or multiple examinations, single lesions or multiple lesions, spanning a single image or multiple images?

The matter of whether or not to include one or multiple subjects is straightforward; the DICOM model allows each instance to encode only information about a single patient. A report about an entire clinical trial would therefore require multiple instances, one or more per subject.

At the opposite extreme though, it would be possible to encode information about findings on single images alone, or for single lesions. Since there is a considerable amount of aggregated information to be encoded, such as the sum of the dimensions of all measured lesions and the overall response, it makes sense to at least make the scope of the template for the document a single examination or time point. For those types of clinical trial protocol that involve multiple modalities, or when progression may be apparent only on other imaging modalities than the primary modality (for example, bone scan detected metastases confirmed on X-ray not seen on the CT of the chest, abdomen and pelvis), it makes sense to make the scope cover at least a single time point without regard to a specific modality or examination. The structure should be flexible to allow some variation in this regard when necessary.

This leaves the question of whether or not to aggregate the results from multiple time points into a single instance. If the purpose of the document is to be a complete longitudinal record of the entire subject’s history at the end of the trial, then for interchange purposes it would be convenient to include all time points in a single document. On the other hand, if the DICOM SR form is to be used internally, within a single software application or shared between cooperating applications, then it may be more appropriate to consider using one DICOM SR instance per time point. This is particularly true in the case of a clinical trial reading paradigm that involves storing and “locking” the results of one time point before the reader is permitted to begin with the subsequent time point. New instances with new content can be created that include only the new content (current time point), or also include the old content (previous time points) either by value or by reference to predecessor documents. The DICOM SR infrastructure allows all of these approaches, so for the time being the issue may be left open, and the template need only make provision for identification of each time point and not be constrained to a single time point.

Similar questions arise with respect to whether a single document should contain the results of only a single reader, or the aggregated responses from multiple readers, perhaps together with the decision of an adjudicator when readers disagree. The DICOM SR infrastructure allows for more than one author (referred to as an observer), and further allows for either individual content items, or a content item and all of its descendents, to have an explicitly defined “observer context” that defines who made the measurements or response assessments. Whilst it may be convenient to have an aggregated report that contains the information from multiple readers, a particular application or system may require them to remain separate, particularly if digital signatures are being applied to an individual reader’s own measurements. Again, it is probably appropriate to leave the template flexible in this regard for the time being, and allow for one or more readers’ results in the same document.

In summary, the template structure should span the possibilities from a single reader’s results for a single time point through all readers’ results for all time points, for a single subject in a single SR document instance.

Looking to the existing DICOM CAD description for guidance, one finds that there are templates used for listing “findings” such as lesions, either on single images or as composite features derived from multiple images. There are templates and codes for describing temporal change, though these are defined primarily in the context of the current examination on which the CAD is performed, and provide a means to describe change, rather than a construct that envisages the encoding of multiple sets of results of the same form but successive in time.

**Figure 2:**
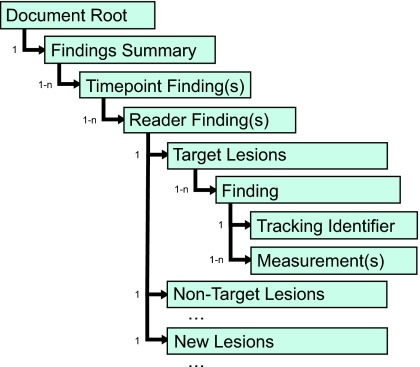
Illustrated is an outline for a clinical trials template organization that allows for one or more time points, one or more readers, and findings (lesions) that span multiple images.

A proposed outline of nested templates that would mimic the existing CAD templates to the extent possible yet introduce the specific concepts of time point and reader is shown in Figure 2. This proposal envisages that for each reader’s report on each time point there will be a single response assessment encoded as the reader’s findings, which will be supported by sub-findings categorized as target, non-target and new lesions. Aggregated information for target lesions, such as the sum of the longest diameter (SLD) or the sum of the product of diameters (SPD) will be encoded in the target lesion container template.

### Findings, descriptions, measurements and coordinates

Evidence document templates generally follow a common pattern of encoding individual measurements and descriptions as “findings”, which may be derived from a single image or set of adjacent slices, or composite features in the sense that they are derived from different findings that are related, such as the same mass identified on two geometrically unrelated radiographic projections. For simplicity, the proposal described here will not address the encoding of composite features, only findings derived from geometrically related images.

Each finding might be composed of:

- a coded identifier specifying the type of finding, which will also serve as the parent node of additional information about the finding,
- a tracking identifier to allow the finding to be referenced from elsewhere in the content tree or referenced externally,
- a list of attributes describing the finding, such as its category (if not implicit from its containment), its anatomic location, its morphological features and its individual response assessment, and
- a list of quantitative measurements of various types, and the spatial coordinates and the images from which they were derived.

### Target lesions

For example, consider how one might encode a RECIST target lesion that is a lung nodule for which the longest diameter has been measured:

<CONTAINER: (,,“Target Lesions”>

> <CODE: (,,“Finding”) = (,,“Nodule”)>

>> <TEXT: (,,“Tracking Identifier”) =

(,,“001”)>

>> <UIDREF: (,,“Tracking Unique

Identifier”) = (,,“1.2.840…”)>

>> <CODE: (,,“Anatomic Site”) = (,,“Apical

segment of left upper lobe of lung”)>

>> <CODE: (,,“Border Shape”) =

(,,“Spiculated”)>

>> <NUM: (,,“Long Axis”) = “13.7”

(mm,UCUM,“millimeter”)>

>>> <SCOORD:(.,“Path”) =

(POLYLINE,133,264,124,273)>

>>>> <IMAGE: = (CT Image,“1.2.840…”)>

In this example, the coded value of the finding is specific such as “nodule”, or “mass” or “effusion”; one could envisage it being more general in those cases where it is not necessary for such information to be determined or recorded, such as “target lesion”. The template could specify both specific and generic context groups for this purpose.

### Tracking Identifiers

Both human-readable as well as globally unique tracking identifiers have been illustrated in this example, and the template should probably require that both always be present. The human-readable identifier is necessary for communication in displays and tabulated results, and the globally unique identifier allows a particular lesion to be referenced persistently from an external document, and tracked over time. Note that in the case of multiple time points, the same human-readable and globally unique tracking identifiers would be used to refer to the same lesion, whether those time points were encoded in the same DICOM SR instance, or in separate instances. It may be desirable to specify a pre-defined list of text values for common naming conventions for human-readable, or a coded list, if consensus could be reached on how lesions should be identified.

### Anatomy and characteristics

Anatomic descriptions may be very general (“chest”), more specific to a region (“lung” or “mediastinum”), or very specific (“apical segment of left upper lobe of lung” or “tracheobronchial lymph node, located near carina”). The use of external terminologies, such as SNOMED and RadLex, both of which describe anatomy thoroughly, allows for very specific descriptions. DICOM templates define subsets of common codes for specific anatomic regions as context groups to reduce unnecessary variation between implementations.

Descriptive attributes such as the shape of the border of a lesion are encoded in DICOM evidence documents as coded name-value pairs, rather than free text descriptions. The existing CAD templates already provide a starting point for a set of descriptors that can be extended as necessary to cover relevant modalities and lesion types, using SNOMED and RadLex codes for consistency. One of the goals of RadLex is to define consistent terminology for use in radiology reports, and hence it will likely provide a sufficiently comprehensive set of descriptors for clinical trials purposes. These descriptive attributes should be optional in the template, however, since many clinical trials do not require that this information be captured because it does not affect assessment of response.

### Non-target and new lesions

In this particular example, a response assessment is not recorded on a per-lesion basis for target lesions, since most criteria aggregate the measurable disease to provide an overall target lesion response assessment. Non-target lesions and new lesions on subsequent time points, on the other hand, do require a categorical response assessment on a per lesion basis, as in the following example:

<CONTAINER: (,,“Non-Target Lesions”>

> <CODE: (,,“Finding”) =

             (,,“Diffuse infiltrate”)>

>> <TEXT: (,,“Tracking Identifier”) =

                (,,“200”)>

>> <UIDREF: (,,“Tracking Unique Identifier”) =

              (,,“1.2.840…”)>

>> <CODE: (,,“Anatomic Site”) =

              (,,“Both lungs”)>

>> <CODE: (,,“Current Response”)=

            (,,“Progressive Disease”)>

### Aggregated response and inference tree

The aggregated information about the measurable disease would be encoded in the template that contained all the target lesions, for example as:

<CONTAINER: (,,“Target Lesions”>

> <contains CODE: (,,“Finding”) =

            (,,“Nodule”)>

>> <has properties TEXT:

        (,,“Tracking Identifier”) = (,,“001”)>…

> <contains CODE: (,,“Finding”) =

            (,,“Nodule”)>

>> <has properties TEXT:

        (,,“Tracking Identifier”) = (,,“002”)>

…

> <contains NUM:

      (,,“Sum of LongestDiameters”) =

      “172.3”(mm,UCUM,“millimeter”)>

> <contains CODE: (,,“Current Response”) =

      (,,“Progressive Disease”)>

There are, in general, two approaches to encoding this type of aggregated information. One may simply list it as contents of the enclosing container node, as in the above example. Alternatively, one can explicitly encode the manner in which the information is derived by using the “inferred from” relationship type. For example:

<CONTAINER: (,,“Target Lesions”>

> <contains CODE: (,,“Current Response”)=

          (,,“Progressive Disease”)>

>> <inferred from NUM: (,,“Sum of Longest

Diameters”) = “172.3”(mm,UCUM,“millimeter”)>

>>> <inferred from CODE: (,,“Finding”)=

                (,,“Nodule”)>

>>>> <TEXT: (,,“Tracking Identifier”) =

              (,,“001”)>

…

>>> <inferred from CODE: (,,“Finding”)=

            (,,“Nodule”)>

>>>> <TEXT: (,,“Tracking Identifier”) =

                (,,“002”)>

…

The latter approach ensures there is no ambiguity about which measurements were used to derive the aggregated information, but adds some complexity to the structure of the tree. The choice of approach depends on whether or not the possibility exists that a container may contain multiple findings, not all of which were used to derive aggregated results. Note in passing that no assumptions should be made about the order of content items at the same level of nesting.

### Geometric measurements

The examples considered so far have emphasized the use of linear distance measurements, since those are used as the basis of RECIST and are currently accepted as endpoints by regulatory authorities. The DICOM SR infrastructure allows for any type of measurement to be encoded, and there are already mechanisms in the standard templates for recording area and volume measurements, which follow the same pattern. For example, a volume measurement obtained by integrating the sum of the areas of a lesion outlined on adjacent slices could be encoded as follows:

<NUM: (,,“Volume”) = “2571.4”

     (mm3,UCUM,“Cubic millimeter”)>

> <has properties SCOORD:

       (,,“Perimeter outline”) =

     (POLYLINE,133,264, …, 133,264)>

>> <selected from IMAGE: =

       (CT Image,“1.2.840….1”)>

> <has properties SCOORD:

       (,,“Perimeter outline”) =

    (POLYLINE,123,259, …, 123,259)>

>> <selected from IMAGE: =

      (CT Image,“1.2.840….2”)>

> <has properties SCOORD:

      (,,“Perimeter outline”) =

    (POLYLINE,101,241, …, 101,241)>

>> <selected from IMAGE: =

      (CT Image,“1.2.840….3”)>

…

For a volume measurement, the boundary of the lesion on each referenced image (CT slice) is encoded as a series of vertices of a closed polygon (indicated by a POLYLINE graphic type with the same start and end point).

The WHO response criteria, which pre-date RECIST and were described for physical measurements rather than measurements on images, made use of the concept of two dimensions, the longest diameter and the greatest perpendicular diameter, and the use of their product as an area approximation (WHO, 1979). Many imaging clinical trials require that this information be recorded, either instead of, or in addition to the RECIST measurement. There is no specific template currently defined in DICOM for WHO measurements. One approach would be to specify the use of two separate measurements using the existing linear distance templates, to encode the long axis (as already suggested for the RECIST measurement) and the additional short axis. The area measurement would be defined by an appropriate code indicating that it is the product of the diameters rather than some other derived area, and could be the parent of the two distances with an “inferred from” relationship. For example:

<CONTAINER: (,,“Target Lesions”>

> <CODE: (,,“Finding”) = (,,“Nodule”)>

>> <TEXT: (,,“Tracking Identifier”) =

        (,,“001”)>

>> <NUM: (,,“Product of Diameters”)=

    “54.8” (mm2,UCUM,“Square millimeters”)>

>>> <inferred from NUM: (,,“Long Axis”)=

      “13.7”(mm,UCUM,“millimeter”)>

>>> <SCOORD: (.,“Path”) = …

>>> <inferred from NUM: (,,“Short Axis”)=

      “4.0”(mm,UCUM,“millimeter”)>

>>> <SCOORD: (.,“Path”) = …

Alternatively, one could simply not record the product of the diameters, since it is easily computed, and is in essence only an intermediate calculation in deriving the sum of the products of the diameters that is recorded in the aggregated results for all target lesions. This approach would avoid the additional level of nesting of linear measurements compared to the RECIST-only case.

### Two and three dimensional coordinates

The coordinate references in SCOORD content items are two-dimensional; they are row and column positions with respect to an image, with fractional (sub-pixel) resolution if necessary. For most DICOM SR use cases this is sufficient, since the information has usually been derived with respect to an image in the first place.

There may be a need to translate image-relative 2D coordinates into patient-relative 3D coordinates. For cross-sectional image modalities such as CT and MRI, DICOM defines a patient-relative Cartesian coordinate space, with respect to an arbitrary origin, which is consistent within a defined frame of reference, identified by a UID, and which typically has the scope of a single acquisition (series or study). For example, a patient will be positioned on the table of the scanner, a reference point identified visually by the operator, and then images acquired, which are encoded with a common frame of reference UID.

Given the 2D row and column coordinates from an SCOORD reference, and the reference to the image on which they are located, one can extract the necessary parameters from the image header (specifically, the 3D patient-relative row and column orientation, the 3D patient-relative location of the top left hand corner (TLHC) voxel, and the pixel spacing), and compute the 3D coordinates. When it is necessary to integrate 2D outlines across contiguous parallel slices, to compute a volume for example, one can derive the reconstruction interval between the centers of slices from the TLHC voxel positions of the referenced images projected onto the normal to the row and column orientation. Note that for such purposes the reconstruction interval should always be computed, since though slices may be parallel and contiguous they may not be equally spaced, and furthermore, the interval may be different from the nominal slice thickness (i.e. there may be a gap or overlap).

It is also possible to copy the necessary position, orientation and spacing parameters from the referenced images and record them somewhere in the DICOM SR instance; this is typically done in the CAD templates, for instance, and the information about all referenced images collected in an “image library” section of the SR document. Whether or not this is worthwhile depends on the intended use of the object. If the images are going to be required for display purposes anyway, copying parameters into the SR object is unnecessary, but if further calculations are to be performed without any other need to retrieve the images, using an image library within the SR may be helpful.

In circumstances in which there is no image from which 2D coordinates were derived, it might be desirable to encode 3D coordinates with respect to some frame of reference directly in the SR object itself. The DICOM SR infrastructure currently lacks a 3D coordinate reference mechanism. There is a supplement to the standard in development that will add a new content item for this, reusing the encoding mechanism already in place for the Radiotherapy Structure Sets and Spatial Fiducials objects currently defined in the standard.

### Non-geometric measurements

The focus so far as been on geometric measurements, which is appropriate since these are the most often accepted as imaging endpoints in clinical trials. However, the DICOM SR mechanisms are equally capable of encoding other quantitative information derived from images. Two notable examples of importance in clinical trials are:

- Hounsfield Units (HU), a measure of tissue density on CT images, and
- Standardized Uptake Values (SUV), a measure of activity on PET images.

For this application, the existing area and volume measurement templates are suitable in terms of their structure, in that they define a measurement type, value and units attached to a defined region of interest in the form of a polygon, circle or ellipse on one or more adjacent slices. Lacking however are:

- the ability to specify a value for a single voxel only (graphic type of POINT),
- a suitable set of concept codes for the category of measurement (HU, SUV),
- a suitable set of modifiers to describe what form of derivation is being performed (mean, median, minimum, maximum, etc.), and
- in the case of SUV, a suitable set of modifiers to describe the type of SUV calculation (i.e. standardization based on body weight (SU-Vbw), lean body mass (SUVlbm), or body surface area (SUVbsa)).

These deficiencies can easily be rectified by defining new templates similar to the existing ones, but dedicated to the purpose, together with appropriate code sets to use for concepts, modifiers and units.

As combined PET-CT technology improves, it is very likely that clinical trials will involve measurements of multiple different parameters of the same region of interest from corresponding registered slices from both modalities, together with geometric measurements derived from the outline of the region of interest on the CT images. The following example might be a description of such a lesion that includes measurements of not only linear distance, but also volume, mean HU density and maximum SUVbw:

<CONTAINER: (,,“Target Lesions”>

> <CODE: (,,“Finding”) = (,,“Nodule”)>

>> <TEXT: (,,“Tracking Identifier”)=

        (,,“001”)>

>>> <NUM: (,,“Long Axis”) = “13.7”(mm,,)>

>>> <SCOORD: (.,“Path”) = …

>>> <NUM: (,,“Short Axis”) = “4.0” (mm,,)>

>>> <SCOORD: (.,“Path”) = …

>> <CODE: (,,“Region of Interest”) = (,,“CT”)>

>>> <SCOORD: (,,“Perimeter outline”) = …

>>> <SCOORD: (,,“Perimeter outline”) = …

…

>>> <has properties NUM: (,,“Volume”)=

        “2571.4” (mm3,,)>

>>> <has properties NUM:

  (,,“Mean Density”) = “-3.6” ([hnsf’U],,)>

>> <CODE: (,,“Region of Interest”) =

        (,,“PET”)>

>>> <SCOORD: (,,“Perimeter outline”) = …

>>> <SCOORD: (,,“Perimeter outline”) = …

…

>>> <has properties NUM:

    (,,“Maximum SUVbw”) = “−3.6” ([g/l,,)>

The challenges in this case are to find a convenient template structure that:

- encodes the outline on each slice once, yet allows for multiple different measurements derived from the same region, and
- establishes a correspondence between a pair of outlines on registered images from two different modalities.

Contrast this approach with the existing geometric measurement templates that make the parent node of the coordinates the measurement itself. Here the inverse approach is taken, in that the concept of a region of interest is defined, which has children that are either one or more outlines, and properties that are measurements derived from those outlines.

An alternative approach would be to use references instead. For example, the existing volume measurement template could be used in the normal manner, and additional measurements other than volume that were derived from the same outlines could reference the content items from another part of the tree, Whilst mechanically feasible, this approach would obscure the fact that that the region of interest was conceptually the same for both measurements.

### Codes for complex measurements

A quantitative measurement may be of a general type that is statistically derived in some manner using some specified technique. For example, an SUV may be computed as the mean value from multiple voxels, and it may be a measure standardized by body weight.

There are two general approaches for encoding the concept that represents such a measurement. A “pre-coordinated” approach is one in which a single code encompasses all that needs to be conveyed. For example, there might be a single code for “maximum SUVbw”. Alternatively, one may use a “post-coordinated” approach, in which there is a single code for “SUV” that is qualified by additional codes, which might include “mean” and “body weight”. The DICOM SR infrastructure allows for either approach, and has a specific relationship, the “has concept modifier” relationship, to allow a child content item to qualify the meaning of its parent.

The choice is more than a mere question of style or preference. The post-coordinated approach allows a query or search for any instance of, say, “SUV”, given a single code with that meaning, without having to be aware of every possible pre-coordinated code that might embody the meaning of “SUV”, such as “SUVbw”, “SUVlbm”, “SUVbsa”, etc. One can see that as the number of dimensions of qualifiers increases there is a combinatorial expansion of the number of codes that must be defined, encoded and explored in a query if the pre-coordinated approach is used.

However, when there are very common patterns of combinations of concepts in common use, and other combinations are uncommon or not meaningful, then the pre-coordinated approach is attractive for its simplicity and convenience. For example, “maximum SUVbw” is very commonly used, whereas other possibilities would be very unusual.

Templates added to the DICOM standard may allow for both approaches, theoretically, but typically define context groups with suggested codes that reflect the most common usage based on available codes from external terminologies, as well as the preference of the constituency defining the templates.

### Definitions of codes

When codes from an external terminology are used in a DICOM SR template, a question arises as to whether or not the meaning of the code is sufficiently precisely defined, and whether or not that meaning is precisely what the authors of the template intend. The argument can be made that the meaning of a relatively imprecisely defined code is refined by the context in which it is used, though this is controversial. The use of post-coordination can also sometimes be helpful in this regard. The choice is something of a balance between the desire to re-use common codes to facilitate interoperability and querying, and the desire for absolute precision.

For example, the context group defined for use with the volume measurement template makes use of a single SNOMED code, G-D705, that has a description of “volume (property) (qualifier value)”, without further elaboration. There is no real definition, either in terms of a descriptive statement or a formal means of deriving this concept from other concepts by relationships (Cimino, 1998). The authors of the CAD templates for DICOM SR thought this insufficient, and defined a set of additional codes for the same template. For example, there is a DCM code 121219 that has a descriptive definition of “a three-dimensional numeric measurement of the bounding region of a three-dimensional region of interest in an image set”. Contrast this with the DCM code 121217 with a descriptive definition of “a three-dimensional numeric measurement that is approximate based on three or more non-coplanar two dimensional image regions”. The desire for additional precision, or distinctly different codes, may arise from the need to encode more than one as properties of the same finding in the same document.

In the context of recording measurements of lesions in clinical trials, the question arises as to whether or not the context of use in a template specifically designed for clinical trials use is sufficient to unambiguously define the more general codes, or whether new codes should be generated. For example, is the use of the SNOMED G-D705 code meaning “volume” sufficient, when it is used as a subordinate content item to a set of target lesions together with a set of spatial coordinates defining the outlines on a set of contiguous transverse CT slices? Or would it be more appropriate to use the more precisely defined DCM code 121219? Or is there a need for a new code that is specifically defined as “a three-dimensional target lesion volume estimate derived by integrating the areas of the semi-automatically detected bounding regions measured on successive contiguous transverse CT slices”?

It may simply be expedient to use for RECIST and WHO-style measurements and corresponding areas and volumes, the following pre-existing SNOMED codes, leaving further interpretation to context:

- G-D705 volume,
- G-A166 area or G-A16A area of defined region,
- G-A185 long axis, and
- G-A186 short axis or G-A195 perpendicular axis.

In a template for clinical trial result reporting, it is unlikely that any ambiguity would arise.

### Response assessment

The paradigm established to categorize overall radiological response is based on a combination of aggregated change in size of measurable disease as well as categorical assessment of non-target and new lesions. A means of encoding response assessment is therefore necessary both at the time point level as well as for each lesion other than target lesions and the first occurrence of new lesions.

The DICOM Chest CAD template already contains a response assessment sub-template that is reusable and allows for specification of both the method and a response category. The template defines codes for both RECIST and WHO. A distinction is made between the current response and the best overall response. Quite likely this template and the context groups and codes will require some revision to make them sufficiently flexible to describe both time point level and lesion level response, as well as to accommodate common variations from the established criteria and to envisage future criteria.

### Splitting and coalescing lesions

Two challenging scenarios to be dealt with between successive time points are when two or more target lesions that are initially separate, grow together, become indistinguishable and must be measured as one, or when one initially single target lesion responds to therapy and divides into two or more lesions. Clinical trial protocols may diverge on how they specify the impact of these scenarios on response assessment, but the primary concern with respect to the DICOM SR encoding is how the lesions on the current and subsequent time points will be identified and tracked with respect to how they are related to the lesions on previous time points.

One possible approach is to specify new Tracking Unique Identifiers, and to explicit specify a relationship to the predecessor UIDs. In addition, it would be desirable to specify some plausible naming convention that recognizes the relationship. For example, if target lesions named “004” and “005” and “006” were to coalesce, they might subsequently be referred to as “004/005/006”.

### Time point identification

Whether or not the root template envisages multiple time points in a single document, or a single document per time point, the need arises to unambiguously identify each time point, and to temporally relate successive time points. Unique identifiers could be assigned to time points to allow them to be referenced from external documents, but human-readable identifiers as well as coded values are necessary to allow time points to be sorted and compared automatically.

The majority of cancer therapeutic trials that involve imaging follow the same paradigm of:

- a pre-registration eligibility assessment phase, which may or may not involve independent review of an imaging endpoint to establish that the patient is progressing on conventional therapy,
- a baseline time point, which may or may not re-use the same imaging examination as the pre-registration progression examination,
- successive time points at pre-scheduled intervals, and
- unscheduled visits when there is clinical progression that may result in imaging being performed.

It would be preferable to define standard codes for each of these time points. However, since the maximum number of time points is theoretically unbounded, one would need to either define an excessive number of codes in advance for the worst-case maximum, or define an algorithm for generating the codes. There is a precedent for using a generated coding scheme in DICOM, in that the UCUM measurements are synthesized rather than pre-coordinated.

For example, one might define codes that mean:

- Pre-study baseline
- Pre-study progression
- On-study baseline
- On-study time point 1 to n
- Unscheduled time point 1 to n

One complication that arises when there is a pre-registration eligibility assessment phase is that the subject may be identified differently in the eligibility phase than in the on-study phase, since the subject is not randomized until they are eligible. This is not likely to be an issue when a single SR document instance is used to encode the entire history of all time points, since the top level patient identification will likely use the on-study randomized subject identifier, and any information acquired prior to randomization will be coerced accordingly. References to images from within pre-registration time point measurements will likely be to images that are labeled with the pre-randomization identifiers, however.

### User identification

As mentioned earlier, the DICOM SR infrastructure supports the notion of “observer context”, which may be either inherited from the top-level header of the document, or specified explicitly at a particular node in the content tree, in which case it is inherited by all the children of that node, unless explicitly overridden. This means that one can specify a container for each reader, and all the contents of that container are defined to have been authored by that reader. The existing observer context templates in the standard are intended for general use by all DICOM SR objects, whether evidence documents or not. They provide attributes to define whom a particular reader is as well as what role they are performing.

It remains for the purposes of clinical trials to specify where in the template explicit definition of observer context is required, and to define appropriate new codes for roles that are relevant. Some potential roles that need to be considered are:

- Eligibility reader 1 to n
- On-study reader 1 to n
- Adjudicator

As was discussed with respect to time point identification, it may be desirable to specify a generated code scheme, rather than to pre-define a fixed maximum number of readers.

The role of the reader should probably be a mandatory attribute in the clinical trials template. The real identity should probably be optional, since it may not be desirable to encode this in documents for interchange outside an organization in some scenarios, though in others it may be required, for example if the SR document is authenticated and digitally signed by the authors.

### Digital signatures

The DICOM standard provides a generic mechanism for digitally signing and time-stamping an entire document or a sub-set of attributes in a persistent, interchangeable manner, using industry standard cryptographic mechanisms and standards. In practice, the deployment of such a mechanism is hampered by the lack of widespread use of a public key infrastructure (PKI) for the validation of such signatures by the recipient.

Furthermore, the signature mechanism is document-oriented, and obtaining multiple, successive, signatures by different authors (readers) contributing content to different components of the content tree is challenging. In such cases, it would likely be necessary to have a separate SR document for each reader and adjudicator.

## Related DICOM Objects

Structured reports and images are not the only types of persistent objects defined in DICOM. Indeed, there are several other types of objects that are relevant to a discussion of graphic annotations and clinical trials.

### Presentation states

Presentation states record the parameters of the steps involved in rendering a grayscale or color image for display. These include a definition of contrast (intensity) transformations, in order to achieve consistency of grayscale or color display on different devices, as well as selection of the area of an image to be displayed (pan, zoom and true size), as well as vector graphics and text to be annotated. They may be automatically generated or captured in response to interactive manipulation and storage of display parameters. They are superior to storing a “screenshot” of a displayed image as a secondary capture image, in that the state may be restored and further interaction performed, in addition to being compact.

Presentation states are specifically not intended for recording any semantics (meaning) associated with vector graphics, but only their rendering. In other words, a presentation state might record the presence of an arrow head composed of line segments pointing at a location, and additional text to be rendered in the same region, but does not record that the arrow head is pointing to the epicenter of a finding or encode the type of finding. As such, while useful for replicating appearance, presentation states are not an appropriate choice for exchanging clinical trials results.

References to presentation states may be included within an SR document, in association with an image reference. Thus the SR may record the meaning of a finding and at the same time point to a presentation state that indicates how an image could be displayed to illustrate the finding in terms of grayscale contrast transformations and displayed area selection. Such a referenced presentation state should not contain graphic or text annotations replicating the SR content, however.

Some workstation and software devices may produce presentation states but not structured reports. In such cases, given sufficient knowledge of how a specific device encodes presentation states in response to user actions, whether documented or reverse-engineered, it may be possible to convert findings encoded in presentation states into findings encoded in structured reports by recognizing certain patterns of graphic annotations and string values in text annotations.

In addition to presentation states for gray-scale and color images, additional presentation states are defined for applying pseudo-color to grayscale images as well as for selecting two sets of images for color fusion, such as for CT-PET fusion.

### Radiotherapy structure sets and regions of interest

The radiotherapy (RT) treatment planning and delivery community has long had an interest in defining coordinates to outline targets, organs to be protected and devices in two and three dimensions. As such, there are mechanisms defined specifically for RT in DICOM that pre-date structured reports and presentation states and that are widely implemented. Their use is largely confined to the RT domain, however, and there is little interoperability with existing general-purpose applications. Unlike presentation states, there is semantic information associated with the RT regions of interest and contours, and conversion into corresponding SR documents would be possible.

### Segmentation objects

A recent addition to the DICOM standard is the segmentation object, which is intended to convey an image-like encoding of how samples in space are classified into categories (segmented). The classification may be binary or probabilistic. There are many applications for segmentation in visualization applications, but this object may also be useful for conveying “probability maps” of human or machine derived lesion boundaries, such as have been proposed for the Lung Image Database Consortium (LIDC) project (Armato, 2004). These segmentation objects can be referenced from within a DICOM SR, and appropriate templates for such references would need to be defined should segmentations prove to be useful for encoding clinical trials results.

### Registration and spatial fiducials objects

There is considerable interest in the spatial registration of image sets from different modalities, such as PET-CT, and from different time points. Both multi-modality fusion and longitudinal comparison are of great interest for oncology clinical trials in particular. Though the automatic rigid or non-rigid registration of two volumes is a non-trivial task, the encoding of such transformations once derived is now supported by DICOM. In addition, since non-rigid registration in particular is often driven by the identification of specific locations within images, fiducials, there is now also a mechanism in DICOM for encoding the location of fiducials.

The use of these mechanisms allows for coordinates of findings defined relative to one image set to be transformed into the coordinate system of a different image set, whether it is from a different modality or a different point in time. One application is for change detection, whether automatically or with human intervention, by propagating the previous location of a finding to a subsequent time point, and reassessing the boundaries of the lesion and then re-computing its size. Another is to facilitate synchronized scrolling and panning of two sets of registered images. The use of the DICOM registration and fiducials objects allows the applications that perform the fiducial detection, registration, volume resampling, lesion propagation and boundary detection to be separated and to be interoperable, in addition to providing a record of the actions perform in a standard format.

### Curves and overlays

There are historical mechanisms that exist within DICOM that are now deprecated or formally retired, and which despite their age are poorly supported by the installed base of devices. These are the curve and overlay mechanisms that allow for annotations to be embedded in a DICOM image itself. The primary reason that the use of these mechanisms is discouraged is that they require that the image obtained from the modality be modified to include the annotation information in the same object. This results in issues with version control as well as unnecessary duplication or modification of the pixel data in order to convey the annotation. In the worst case, if annotated images are available from an older device or software tool that uses one of these mechanisms, the information can be extracted and converted into a presentation state or a structured report referencing the original image. The use of curves and overlays in the image object for encoding clinical trials results should be strongly discouraged.

## Implementation

### Tools

The full text of the entire DICOM standard is somewhat daunting, to say the least. Fortunately, there is a wide variety of free, open source and commercial toolkits in various popular languages for the majority of popular operating systems available to anyone interested in implementing DICOM. Such tools not only support the encoding, display, transfer, query and storage of images, but also in many cases have specific support for DICOM Structured Reporting as well. Various tools allow for the programmatic construction and parsing of SR documents, provide user interface components and applications for storing, querying, retrieving, authoring, editing and rendering of them, and often provide support for trans-coding into intermediate or rendered forms such as XML, HTML or PDF.

Typically, the developer of a quantitative imaging application for use in clinical trials will be faced with the need to encode in a persistent form any annotations created by a user or the software, and to reload such annotations and edit and re-save them. They will often use either a programmatic interface inherited from the toolkits from which the application is constructed, or develop some completely new means such as their own proprietary file format or database structure in which to store the information. It is relatively straightforward to adapt such code to make use of the programmatic interface of a DICOM toolkit that is SR-aware to achieve the same objective. Developers should rarely, if ever, have to hand-code DICOM encoding, parsing and network code, unless they are motivated by a truly masochistic desire to develop a new toolkit from scratch. Almost certainly, any medical imaging application will already be using a DICOM toolkit in order to gain access to the image data in the first place.

In general, such toolkits make the parsed or created SR content tree available as some sort of abstract tree object, with methods for traversing and searching the tree and extracting individual nodes and attributes. The techniques are identical to those used when manipulating a Document Object Model (DOM) (W3C, 2005) as is common within scripts used in web browsers and XML processing applications, or manipulating the tree model that underlies a typical browser component in a user interface framework (such as a the TreeModel that backs a JTree in Java).

Another approach is to separate the DICOM SR encoding from the application, and make use of adapters or translators that take the proprietary file format saved by the application, or which perform a query on proprietary tables in the database containing the information, and construct a DICOM SR object, or vice versa. Wrapping legacy applications in such a manner may be an easier and more expedient means of achieving interoperability than changing the application itself. A common approach is to extract and translate the proprietary format into some equivalent XML representation, apply an eXtensible Stylesheet Language Transformation (XSLT) (W3C, 2006a) to convert it into an XML equivalent of a DICOM SR, and then transform that in turn into a binary DICOM encoding. Indeed, many contemporary annotation file formats used within both commercial and academic medical imaging software tools already make use of an XML representation, using a proprietary DTD or Schema, and it is usually relatively straightforward to transform them to and from DICOM SR using XSLT, whether the format is documented or can be reverse engineered. For example, the LIDC uses a simple, documented, XML format for outlining nodules on chest CT, which is easily trans-coded into DICOM SR to make it potentially interoperable with commercial Chest CAD applications that use standards.

Though any mention of specific tools and tool-kits will necessarily become out-dated, and a review of commercial applications is inappropriate, it may be helpful to the reader to describe several examples of open-source software. The OFFIS dcmtk toolkit not only provides a high quality multi-platform implementation of DICOM encoding and network functions in C++, but also contains specific tools for handling DICOM SR, including utilities for trans-coding DICOM to XML, as well as for rendering SR to HTML (OFFIS, 2007). The DICOMScope tool from the same group was developed to demonstrate some of the concepts for editing and viewing DICOM SR documents (OFFIS, 2001). The PixelMed Java DICOM toolkit by the author includes similar tools, as well as classes for modeling SR trees, browsing them and some examples of display capability, such as for the application of mammography CAD marks to images (PixelMed, 2007). Limited DICOM SR capability has also been integrated into clinically useful workstation software as opposed to toolkits or demonstration software. The OsiriX application for MacOS X is a notable example, which can store and display regions of interest as well as human-readable reports as DICOM SR objects (Rosset, 2007).

## Relationship to Other Standards

### XML

There are a variety of other efforts related to the standardization of the encoding of structured healthcare information, the most notable of which are XML-based. It is quite likely that a completely new standard for encoding radiological images and related information written in the 21st century would provide XML as an underlying encoding mechanism for everything except the bulk binary data. However, DICOM standardization began in the mid-1980s, with the first modern version being released in 1993, before the XML specification was published, and well before it became popular. A binary tag-value pair encoding form was used in DICOM, similar to that used in the Tagged Image File Format (TIFF) that was being developed for the graphic arts industry at about the same time. Other image formats like JPEG and GIF, as well as the more recent PNG and JPEG 2000, all of which also use binary encoding of header attributes, are not going to be replaced just because they are not XML-based. Similarly, the large installed base of DICOM devices precludes a change at this time.

During the development of DICOM SR in the mid to late-1990s, consideration was given to using XML as an alternative to a pure binary DICOM encoding. This approach was rejected in favor of making use of the existing available DICOM tools and libraries that were already deployed in commercial medical devices. XML tools were not at that time as widespread, robust or accessible through standardized interfaces. The question of now standardizing an “alternative” XML encoding of the DICOM SR structure is revisited from time to time in the various DICOM working groups. The counter-argument to maintain interoperability with the installed base of devices and software prevails. In addition, the specific form of XML encoding to standardize would be heavily dependent on the intended use, and would likely require an additional choice to be made from many additional standards that are layered on top of XML for specific purposes.

In the absence of a standard alternative encoding of DICOM SR in XML, there are still many reasons to use XML as an intermediate form within applications. An implementation pattern for applications that render DICOM SR for human consumption is to transform the binary encoded content tree first into an XML form, either literally or into an object model or series of events, then apply XSL transformations to create HTML, then view that in a browser. The choice of intermediate XML form is very dependent on both the needs of the application and assumptions about the structure of the content. For example, if it is known that the content is only about individual lesions and their response evaluation, one could construct XML fragments of the form:

<Lesions>

  <TargetLesion name= “001” diam

        eter=“37.3”/>

  <NonTargetLesion name= “200”

      response=“PD”/>

</Lesions>

This sort of form is typical of the ad hoc XML formats that individual developers devise to solve specific problems without any concern for interoperability beyond their immediate domain; it is easy to write with a text editor, easy to extend, and is to some extent self-describing, in that the element names are human-readable and their meaning is implicit, perhaps dangerously so. Note in this example that the units of distance are implicit, and hence not self-described.

Such a compact and application-specific representation may not be as useful for general-purpose applications that use codes from controlled vocabularies for concepts and values. Here is an alternative example that might be a more “literal” translation of a DICOM SR content tree and which explicitly preserves the use of coded terminology:

<Container csd=“DCM” cv=“121070”

      cm=“Findings”>

  <Code csd=“DCM” cv=“121071”

        cm=“Finding”>

    <Value csd=“” cv=“”

      cm=“TargetLesion”/>

    <Code csd=“” cv=“” cm=“Name”>001

        </Code>

    <Num csd=“SNM3” cv=“M-02550”

      cm=“Diameter”>

    37.3

    <Unit csd=“UCUM” cv=“mm”

      cm=“millimeter”/>

  </Num>

</Code>

<Code csd=“DCM” cv=“121071” cm=“Finding”>

  <Value csd=“” cv=“”

      cm=“NonTargetLesion”/>

<Code csd=“” cv=“” cm=“Name”>200</Code>

<Code csd=“” cv=“” cm=

      “Response Assessment”>

    <Value csd=“” cv=“” cm=

      “Progressive Disease”/>

    </Code>

  </Code>

</Container>

There are many choices for encoding concepts drawn from a coded terminology. Rather than use attributes of an XML element as above, one might call out the coding scheme designator, code value and code meaning as separate elements, as in this example:

<Unit>

  <CodingSchemeDesignator>UCUM

      </CodingSchemeDesignator>

  <CodeValue>mm</CodeValue>

  <CodeMeaning>millimeter</CodeMeaning>

</Unit>

XML namespaces are an alternative to explicitly representing the coding scheme, code value and code meaning as XML attributes or elements. Once can declare an XML namespace that represents the coding scheme, or some designated subset or version of it. For example, if an XML document were to declare the namespaces “dcm”, “snm3” and “ucum”, then parts of the earlier example might be encoded as any one of the following possibilities:

<dcm:121071>

  <snm3:M-02550>37.3</snm3:M-02550>

  <ucum:mm/>

</dcm:121071>

<dcm:Finding>

  <snm3:Diameter> 37.3</snm3:Diameter>

  <ucum:millimeter/>

</dcm:Finding>

<dcm:121071 cm=“Finding”>

  <snm3:M-02550 cm=“Diameter”>

      37.3</snm3:M-02550>

  <ucum:mm cm=“millimeter”/>

</dcm:121071>

Evaluation of such approaches raise the issue of whether or not the string representation of code values from the coding scheme may be encoded as XML elements, whether or not self-describing element names are defined by the name space, either by the coding scheme or elsewhere, and whether or not to encode a human-readable meaning in the message. Regardless, namespaces are a fundamental mechanism used by XML-based standards to enhance interoperability. In particular, they allow a single document to make use of elements whose meaning is defined elsewhere by multiple different standards.

### HL7 CDA

The Clinical Document Architecture (CDA) is a standard defined by the Health Level Seven (HL7) organization (HL7, 2004) for the purpose of interchanging persistent XML-encoded clinical documents. It allows for both structured and unstructured as well as coded content, and provides a means of referencing or wrapping multimedia content. There are well-defined meta-data elements for managing the document, such as to be able to clearly identify the author and subject of the document. CDA documents are required to be human readable, in the sense that they must contain narrative content equivalent to any structured content present. The architecture makes use of the Reference Information Model (RIM) as well as data types being defined for the HL7 Version 3 (V3), but is not dependent on deployment of HL7 V3 for interchange. That is, CDA documents can be persistent and standalone, and be interchanged through other mechanisms than HL7.

HL7 and DICOM have worked together to harmonize the CDA and SR efforts and to avoid gratuitous incompatibilities, but no mechanism of bi-directionally trans-coding SR to CDA with full fidelity is yet formally defined by either group, and may never be.

CDA contains a means of encoding regions of interest in referenced images that provides similar mechanisms to those present in DICOM, as well as similar mechanisms for encoding concepts from controlled vocabularies. Similar graphic element types are used and the same mechanism is used for referring to two-dimensional coordinates as column and row pixel offsets from the top left hand corner of an image. The following is a simplified example:

<RegionOfInterest>

  <code code=”POLY”/>

  <value>133 264 124 273</value>

  <entryRelationship typeCode=”SUBJ”>

    <ObservationMedia>

    <value xsi:type=”ED”

      mediaType=”application/dicom”>

      <reference value=”image.dcm”/>

       </value>

    </ObservationMedia>

  </entryRelationship>

</RegionOfInterest>

Such a coordinate reference in a CDA might occur within an observation, such as the following:

<Observation>

  <code code=”81827009”

    code System=”2.16.840.1.113883.6.96”

    codeSystemName=”SNOMED CT”

    displayName=”Diameter”/>

…

</Observation>

CDA follows the HL7 V3 practice of using Unified Medical Language System (UMLS) Concept Unique Identifiers (CUID) as code values, rather than the traditional SNOMED code values that DICOM uses, but these can be mapped to one another with no information loss. The code system, which DICOM refers to as the coding scheme designator, is encoded as a globally unique identifier, specifically as an OID (Object Identifier), which is the same as a DICOM Unique Identifier (UID); these can also be mapped with full fidelity to and from the DICOM representation, and DICOM also has added specific mechanisms to encode the mapping of standard or private coding scheme designators to UIDs in a document instance.

### Ontologies and W3C image annotation efforts

This paper will not attempt to define the meaning of the word “ontology” in a general philosophical sense or in the more specific context of medical informatics. It suffices to say that considerable attention is being devoted to the matter of knowledge representation, and that various standards and tools exist for the interchange of “ontologies” and the encoding of instances of entities in the real world classified by such ontologies. A prominent example is the Web Ontology Language (OWL) (W3C, 2004a), which is in turn layered on top of the Resource Description Framework (RDF) (W3C, 2004b), and uses an XML encoding with an extensive use of namespaces. The intent of RDF is to describe information contained in properties and property values about things called resources, which can be identified or referenced. OWL uses RDF to encode definitions of classes of things and the relationships between them. For example, an ontology of lesions and their characteristics and quantitative measurements could be devised and exchanged in OWL format, and instances of entities in the real world modeled by such an ontology could be encoded. It is important to distinguish between the encoding of the ontology itself, however, from instances that are classified by that ontology, though both may be encoded in XML.

The classes and relationships and cardinalities in such an OWL model would be analogous to content items defined in any particular DICOM SR template, and likely could be mapped with high fidelity. One advantage of having an OWL model is that it allows for general-purpose knowledge representation tools such as Protégé (Gennari, 2002) to be used to generate and maintain the ontology. Another advantage is that it enables the exchange of information more generally both within and beyond the healthcare and biological research domains. Indeed, should the concept of the “semantic web” (Berners-Lee, 2001) prove successful, the re-use of consumer industry tools for image and region of interest annotation would be possible. An example of a general-purpose ontology-based image annotation tool is PhotoStuff (MindSwap, 2003), which can to be used to create and edit image-related instances of an ontology developed in Protégé and encoded in OWL.

Because of the generic nature and the use of namespaces, the form of the XML encoding when using such a general-purpose approach may be more obscure to the human observer, but the meaning may be more easily extracted by software tools, particularly when classes of descriptors that are widely adopted are used, such as are proposed by the W3C for image annotation (W3C, 2006b). Here is an edited and simplified example of an instance of a region that might be created and stored by such an application:

<rdf:RDF

  xmlns:j.0=

    “http://www.w3.org/2003/12/exif/ns/”

  xmlns:rdf=

  “http://www.w3.org/1999/02/22-rdf-syntax-ns#”

  xmlns:rdfs=

  “http://www.w3.org/2000/01/rdf-schema#”

  xmlns:j.2=

    “http://www.w3.org/2004/02/image-regions#”>

<rdf:Description

    rdf:about=”file:/imagedcm#region1”>

   <j.2:coords>216,299 155,406 …

      </j.2:coords>

   <rdf:type rdf:resource=”http://www.w3.org/2004/02/imageregions#Polygon”/>

   <rdfs:label>Lesion 001</rdfs:label>

  </rdf:Description>

</rdf:RDF>

This approach can be extended to add more descriptive metadata within the rdf:Description element, perhaps elements specific to medical images and clinical trials, using namespaces that corresponded to the appropriate vocabularies, the choices amongst which would be constrained by the appropriate ontology. This is achieved within the tool by importing the ontology in a standard format, at which time the descriptors in the ontology become available for use to describe images and regions, within the constraints of the allowable cardinalities and relationships defined in the ontology.

## Conclusions

Clinical trials of therapeutic agents are increasingly making use of imaging-derived biomarkers as endpoints, necessitating the interchange of quantitative and categorical information that is image-related. It is clearly important to reuse technology and devices designed for use in the healthcare imaging environment, where the use of DICOM is ubiquitous.

The DICOM Structured Reporting mechanism was developed specifically to address the encoding of such information and is being deployed to meet the needs of clinical CAD and other applications that capture information derived from images. Many of the templates that have been defined for these applications are equally appropriate for use for clinical trials, or provide guidance for development of new templates.

Given the relative maturity of the various standards, documents, tools and development efforts described, the author recommends that DICOM SR be chosen as the primary encoding mechanism for the delivery and interchange of clinical trials results. Further, standard templates should be developed for specific types of trials.

Interoperability with the XML-based world of HL7 CDA and the ontology-orientated semantic web will be important, and harmonization with such efforts during the development of clinical-trial templates for DICOM SR is clearly required. The DICOM SR clinical trial templates would then serve as the basis for trans-coding into other standards or forms to take advantage of more generic tooling.

## Figures and Tables

**Table 1:** Template TID 1400—Linear Measurement

**Row**	**Nesting Level**	**Relationship with Parent**	**Value Type**	**Concept Name**	**Value Multiplicity**	**Requirement**	**Condition**	**Value Set Constraint**
1			NUM^a^	DCID (7470) “Linear Measurements”^b^	1	M		UNITS = DCID (7460) “Units of Linear Measurement”^c^
2	>	INFERRED FROM	SCOORD^d^	(121055,DCM, “Path”)^e^	1	M		
3	>>	R-SELECTED FROM^f^	IMAGE		1	MC^g^	XOR Row 4	
4	>>	SELECTED FROM	IMAGE		1	MC	XOR Row 3	

Note the following explanations of the features of this template:

^a^ This template is used by other templates by inclusion; the root node of this sub-template is a single numeric measurement content item (of type NUM), which consists of a coded concept name, a single numeric value and a coded unit; only one of these content items may be present, as indicated by a value multiplicity of 1, and it is required be present, as indicated by the requirement of mandatory (M).

^b^ The concept name used for the numeric content item shall be one of a set of codes list in the context group DCID 7470, which contains a list of linear measurement types. See Table 2.

^c^ The units of the numeric content item shall be one of a set of codes list in the context group DCID 7460, which contains a list of linear measurement types. See Table 3.

^d^ Additional textual description in the standard, not included in the tabular representation, constrains the form of the SCOORD to be either a POLYLINE, CIRCLE or ELLIPSE.

^e^ The concept name for the spatial coordinates content item is a single coded value, which happens to be from the DICOM terminology.

^f^ The image to which the coordinates specified in the parent content item apply may either be present as a child item (as specified by row 4), or a reference to another location elsewhere in the content tree (as specified by row 3 and indicated by the “R-” prefix to the relationship, which means “reference”); in the latter case the structure would not be a tree but a directed acyclic graph.

^g^ Rows 3 and 4 illustrate the use of a mandatory conditional (MC) requirement with a formally described exclusive-or condition and a multiplicity of one, in order to specify that a content item instantiating one or the other row shall be present but not both.

**Table 2:** CONTEXT GROUP CID 7470–LINEAR MEASUREMENTS^a^

**Coding Scheme Designator**	**Code Value**	**Code Meaning**
SRT b	G-A22A	Length
DCM c	121211	Path length
DCM	121206	Distance
SNM3 d	G-A220	Width
SRT	G-D785	Depth
SNM3	M-02550	Diameter
SNM3	G-A185	Long Axis
SNM3	G-A186	Short Axis
SRT	G-A193	Major Axis
SRT	G-A194	Minor Axis
SRT	G-A195	Perpendicular Axis
SNM3	G-A196	Radius
SRT	G-A197	Perimeter
SNM3	M-02580	Circumference
SRT	G-A198	Diameter of circumscribed circle
DCM	121207	Height

Note the following explanations of the features of this template:

^a^ This context group is declared in the standard to be extensible, which means that additional codes may be added to it as the need arises.

^b^ SRT indicates that the code is from SNOMED.

^c^ DCM indicates that the code is from DICOM.

^d^ SNM3 also indicates that the code is from SNOMED; the use is historical since the designator was defined and used for Version 3 of SNOMED; terms adopted from any subsequent version of SNOMED will use a designator of SRT.

**Table 3:** Context Group CID 7460–Units of Linear Measurement

**Coding Scheme Designator^a^**	**Code Value**	**Code Meaning^b^**
UCUM	cm	centimeter
UCUM	mm	millimeter
UCUM	um	micrometer

Note the following explanations of the features of this template:

^a^ The Unified Code for Units of Measure (UCUM) (Schadow 2005) is used for all units in DICOM SR templates. UCUM describes a formal syntax for synthesizing units to measure any physical property.

^b^ The meaning is not normative, and alternative spellings of units may be used that are appropriate to the local language, as is defined elsewhere in the standard.
